# Tracking functional network connectivity dynamics in the elderly

**DOI:** 10.3389/fnins.2023.1146264

**Published:** 2023-03-20

**Authors:** Kaichao Wu, Beth Jelfs, Seedahmed S. Mahmoud, Katrina Neville, John Q. Fang

**Affiliations:** ^1^Department of Biomedical Engineering, College of Engineering, Shantou University, Shantou, China; ^2^School of Engineering, Royal Melbourne Institute of Technology University, Melbourne, VIC, Australia; ^3^Department of Electronic, Electrical and Systems Engineering, The University of Birmingham, Birmingham, United Kingdom

**Keywords:** aging, dynamic functional network connectivity, graph theory, mnemonic discrimination ability, functional integration and segregation

## Abstract

**Introduction:**

Functional magnetic resonance imaging (fMRI) has shown that aging disturbs healthy brain organization and functional connectivity. However, how this age-induced alteration impacts dynamic brain function interaction has not yet been fully investigated. Dynamic function network connectivity (DFNC) analysis can produce a brain representation based on the time-varying network connectivity changes, which can be further used to study the brain aging mechanism for people at different age stages.

**Method:**

This presented investigation examined the dynamic functional connectivity representation and its relationship with brain age for people at an elderly stage as well as in early adulthood. Specifically, the resting-state fMRI data from the University of North Carolina cohort of 34 young adults and 28 elderly participants were fed into a DFNC analysis pipeline. This DFNC pipeline forms an integrated dynamic functional connectivity (FC) analysis framework, which consists of brain functional network parcellation, dynamic FC feature extraction, and FC dynamics examination.

**Results:**

The statistical analysis demonstrates that extensive dynamic connection changes in the elderly concerning the transient brain state and the method of functional interaction in the brain. In addition, various machine learning algorithms have been developed to verify the ability of dynamic FC features to distinguish the age stage. The fraction time of DFNC states has the highest performance, which can achieve a classification accuracy of over 88% by a decision tree.

**Discussion:**

The results proved there are dynamic FC alterations in the elderly, and the alteration was found to be correlated with mnemonic discrimination ability and could have an impact on the balance of functional integration and segregation.

## 1. Introduction

Aging has a profound influence on the brain's structure and function at both local and global scales. These effects are responsible for decreased mental and physical fitness (Cole et al., 2018) and increased risk of neurodegenerative diseases such as Alzheimer's disease (Abbott, 2011), or Parkinson's disease (Dennis and Thompson, 2014; Reeve et al., 2014). Functional magnetic resonance imaging (fMRI) is a powerful and efficient, accessible and non-invasive tool, which has been extensively used to reveal neural mechanisms engaged in the normal aging process. It has also contributed greatly to elucidating the role that aging plays in the decline of brain function [e.g., the cognitive (Uddin et al., 2017) or motor function (Thomason et al., 2008)]. More precisely, resting-state fMRI studies have frequently reported altered connectivity both within-network and between-network. In human aging the findings encountered include: the functional connectivity (FC) decreases within higher-order networks and segregation of networks diminishes with advancing age. For example, within the default network, the salience network, and the frontoparietal control network, FC has been reported to be reduced (Fjell et al., 2016; Grady et al., 2016). This FC alteration could be a sign of neural or functional network reorganization, however, these findings rely on the static functional network connectivity analysis (SFNC). One potential limitation of SFNC is the theoretical assumption that the FC exhibits a constant state during a rest MRI period. This means that the fine-grained temporal evaluation of resting state has been neglected, and the flexibility of the functional network reorganization cannot be assessed.

Recently, with the advances in understanding of the temporal resolution of resting-state fMRI, the interest in how normal human aging affects the time-varying or dynamic functional network connectivity (DFNC) has increased (Calhoun et al., 2014). For instance, the loss or decline of FC dynamics has been wildly found in the elderly adult group (Schaefer et al., 2014; Chen et al., 2017). This temporal variation of FC reflects the network flexibility necessary for brain function response, which fits our intuitive perception of the elderly who have the loss of physical flexibility. In contrast with SFNC, an advantage of DFNC is that it allows the fluctuation of FC, within or between the brain functional networks, over short periods to be observed. Identification of the FC fluctuation patterns allows the brain's FC state profile to be identified. Following which, features characterizing the FC dynamics, such as the transition trajectories between distinct brain states (Allen et al., 2014; Vidaurre et al., 2021) can then be used to interpret brain behaviors.

Given such a capacity, DFNC has been increasingly applied to brain aging. For instance, the DFNC method has demonstrated that the FC dynamics degenerate in normal aging. This degeneration is reflected by the lower switching rate between brain states within salience network (Snyder et al., 2021) and default network (Xia et al., 2019), as well as by the decreased connectivity flexibility in the right middle frontal gyrus (Yin et al., 2016). The FC dynamics has also been demonstrated to correlate with cognitive ability (Xia et al., 2019). Studies using DFNC methods have revealed other opinions regarding dynamic FC. For example, FC dynamics is usually characterized by the switching rate of connectivity states, which is defined as the rate at which a state transitions between potential functional brain states over a certain period. However, in a study investigating the human brain across the lifespan, for example, the switching rate of brain state was observed to have no difference between different age groups (Viviano et al., 2017). These distinct results are possibly due to differences in the implementation method and the data samples. While the results are not consistent, all these collected findings imply that the DFNC analysis is a promising method for providing insight into human aging neuromechanisms from multiple views and means.

Therefore, in this research, we explore the brains of two age-different groups with the DFNC method, to track the FC dynamics in the elderly over the MRI scan and to investigate the relationship between dynamic FC and age. Overall, we expect that the study of DFNC can reveal and track the change in flexibility of function coordination and interaction in the elderly, and this alteration can facilitate brain age estimation at an individual level. This research also has the potential to form the basis for further investigations which may provide a deeper understanding of brain changes and aging. This could offer clues to the relationship between brain maturity and brain behaviors as well as age-induced diseases.

Specifically, the resting-state functional MRI data from 34 younger adults aged 19–22 and 28 elderly adults aged 60–80 have been tested by an implemented DFNC analysis pipeline. The fMRI data is used to identify the intrinsic connectivity networks (ICNs), from which the brain states are estimated and the dynamic features extracted. The alterations in FC dynamics caused by aging were examined, and the power of dynamic features in individual age prediction was evaluated in this framework. In addition, we have also discussed the relationship between dynamic features and mnemonic discrimination ability and the dynamic balance of functional integration and segregation in healthy aging.

## 2. Materials and methods

### 2.1. fMRI acquisition

Resting-state scans were obtained from the University of North Carolina samples at Greensboro^1^ after request, without any rights conflicts. The participants were 28 elderly adults (61–80 years old, mean age ± standard: 69.82 ± 5.64; 20 female) and 34 young (18–32 years old, mean age ± standard deviation(SD): 22.21 ± 3.65; 20 female). Participants were instructed to lie motionlessly in the scanner and stay awake with their eyes open. All functional images were collected using a Siemens Trio 3.0T scanner with a 16-channel head coil and the following recording parameters: 32 slices with 4.0 mm thickness and no skip, time of echo = 30 ms; time of repetition (TR) = 2,000 ms; flip angle = 70, field of view = 220 mm, matrix size = 74 × 74 × 32 voxels, 300 volumes in 10 min.

### 2.2. fMRI data preprocessing

The data for each participant has 300 measurements recorded over 10 min. The first five volumes of each scan were discarded to allow for magnetic stability and thus to generate a steady blood oxygenation level-dependent activity signal. The functional data was then processed with the following steps:

1. Realignment to correct head motion (see Section 2.3 for verification details).
2. Slice time correction.
3. Outlier identification.
4. Normalization (normalize to 3 mm MNI space using a templates from the SPM software package; Ashburner and Friston, 2005).
5. Spatial smoothing with a Gaussian kernel of 8 mm full-width at half-maximum (FWHM).

The processing pipeline was executed using the CONN toolbox (Whitfield-Gabrieli and Nieto-Castanon, 2012).

### 2.3. Verification of head motion correction

To verify there was no significant head movement in the data, for each participant the individual mean and maximum framewise displacements (FD) (Power et al., 2012) were calculated. As the participants with large outlier scans have been removed from the raw data, none of the available participants had head motion greater than 0.5 mm. No significant group difference in FD was observable when comparing the final sample of 28 old adults and 34 young adults (*p* = 0.92).

### 2.4. Static functional network connectivity analysis

To assess static connectivity, pairwise Pearson correlations were computed over the entire timeseries and then Fisher's Z-transformed. Group ICA-based was used to produce brain parcellations according to the same procedure as described in Section 2.5.1. This calculation resulted in correlation coefficients per participant, which represent the connectivity strength between the given ICNs. Then, the static connectivity matrices were averaged across the young and elderly adult groups.

The difference in static connectivity between the young and elderly groups was evaluated through a two-sample *t*-test (a significance level of *p* < 0.05). The correction for multiple comparisons was applied using false discovery rate (FDR)-correction to determine statistical significance at *p* < 0.05 (Benjamini and Hochberg, 1995).

### 2.5. Dynamic functional network connectivity analysis

**Figure 1:**
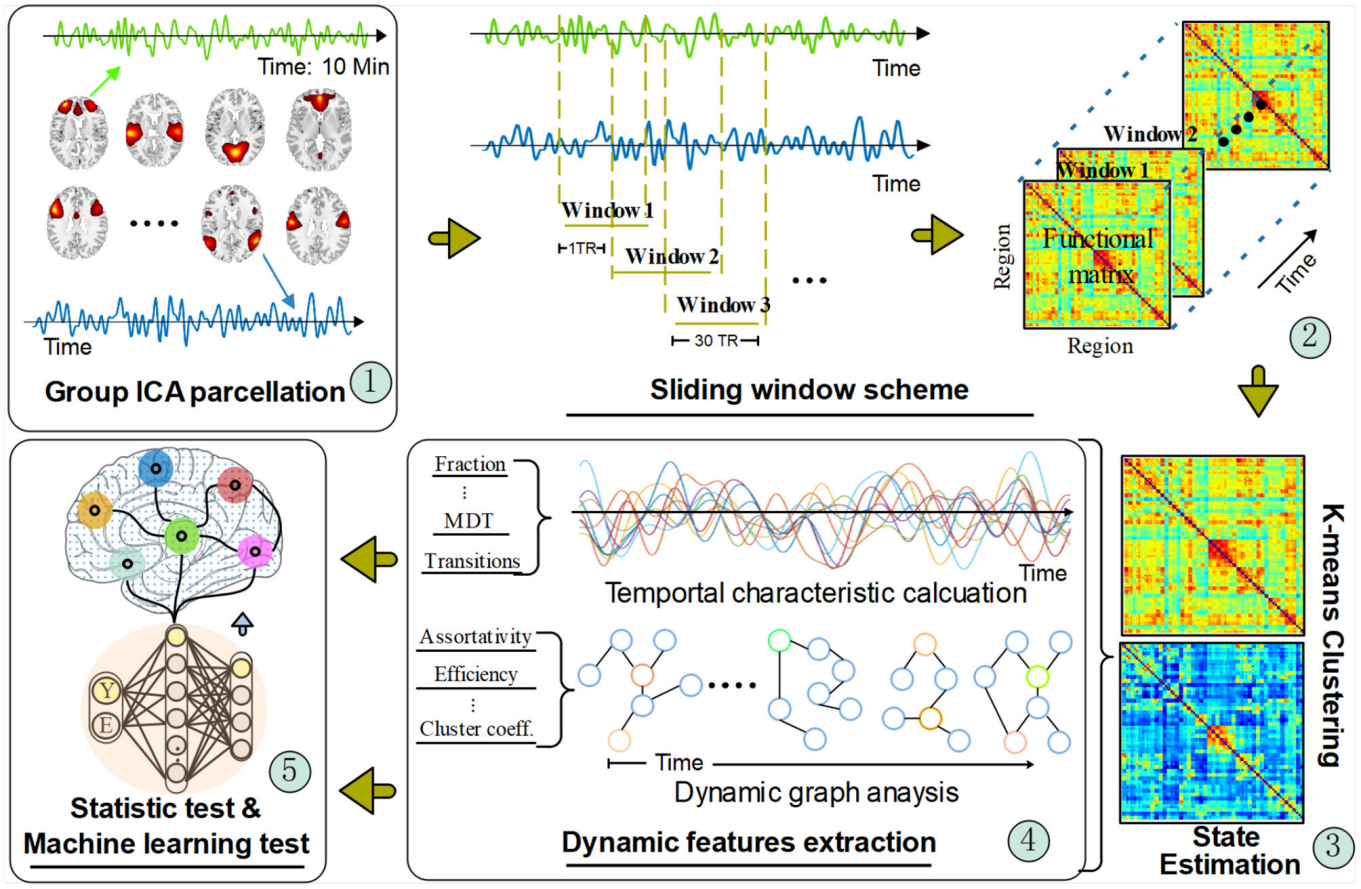
The dynamic functional connectivity analysis pipeline. The timeseries signal was extracted from the network regions recognized from the group ICA parcellation method. Then, the regional timeseries were decomposed with a sliding window scheme for a time-varying function network connectivity (FNC) estimation. Those FNC matrixes were fed into a clustering algorithm to obtain different transient brain states by forming a cluster centroid. After that, two types of dynamic features were calculated based on the acquired transient states and temporal signals. Finally, statistical and machine learning methods were applied to verify the extracted dynamic FC features.

Figure 1 shows the framework of our DFNC approach. Specifically, there are five main steps in this pipeline:

1. Group independent component analysis (ICA) parcellation for intrinsic connectivity network (ICN) recognition,
2. Sliding window cross-correlation,
3. Clustering analysis for brain state estimation,
4. Dynamic feature extraction,
5. FC dynamics examination *via* statistics and machine learning tests.

Details of each step are provided in the following sections.

#### 2.5.1. Step 1: Group independent component analysis parcellation

Group ICA was performed in order to parcellate the brain into various functional networks. Following the recommendations from previous studies (Allen et al., 2014; Abrol et al., 2017; Xia et al., 2019), the number of components that can functionally parcellate the brain was predefined at 100. The configuration for the group information-guided ICA algorithm was developed according to the detailed description provided by Salman et al. (2019). In particular, we adopt the two-stage Principal Component Algorithm (PCA) to preserve the components that account for the most variance. In the first stage, each participants' functional data was decomposed into 120 principal components (PCs), and the PCs of all participants were concatenated across time and then further reduced to 100 in the second stage. Finally, the infomax algorithm, from the ICASSO software package (Himberg and Hyvarinen, 2003), was used with 20 repeats to find steady independent components (ICs). After back reconstruction, the participant-specific spatial maps and corresponding time courses can be obtained. Three methods were employed to detect the ICNs from potential functional networks:

1. The spatial activation maps from the ICs were visually inspected to identify if they match the large-scale functional network locations from previous studies (Di and Biswal, 2015; Kim et al., 2017) and anatomical brain regions.
2. The multiple regression method was used to select ICs whose spatial pattern matches with the existing functional networks template given by:

(1) $$\begin{array}{l} Y=\beta_{1}X_{1}+\beta_{2}X_{2}+\cdots +\beta_{n}X_{n}+\varepsilon , \end{array}$$

where *Y* is the collection of the spatial vector of template ICNs, *X*_*i*_ represents the spatial vector of the *i*-th IC and β is the regression coefficient. The regression analysis is used to select the ICs closest to the functional network template spatially (the first rank of β), and the calculation is done by least-squares estimation.
(3) The power spectrum of the ICs was checked to see if it follows a low-frequency peak and a high-frequency steady pattern (the time courses of ICs are characterized by high dynamic range) (Griffanti et al., 2017).

Following the practice presented in Tu et al. (2019) and Bonkhoff et al. (2020), before passing the ICNs to the subsequent steps of the DFNC pipeline, additional post-processing of the time courses of all included ICNs was performed. The post-processing involved (a) linear, quadratic, and cubic detrending, (b) regressing out motion parameters (six realignment parameters and their first temporal derivatives), (c) low-pass filtering with a high-frequency cut-off of 0.15 Hz (to retain only BOLD-related signal fluctuations; Calhoun et al., 2001), and (d) despiking using 3D despike. These actions ensure artifact noise has minimal impact on the signal analysis.

#### 2.5.2. Step 2: Sliding window cross-correlation scheme

In the second step a sliding window is used to segment the timeseries of the ICNs into sub-fragments. For each time window the correlations between the ICNs during that window were calculated. There is no consensus in terms of the window size and the length of the sliding step. However, prior studies provide evidence that a window size between 30s and 60s enables successful estimation of DFNC giving an appropriate balance between accurate calculation of the correlation and the ability to detect time variations in the ICN timeseries (Hindriks et al., 2016; Liegeois et al., 2016; Preti et al., 2017). Thus, in our experiment, we opted for the common parameter settings, where the width of the window is 22 TR time (Kim et al., 2017; Bonkhoff et al., 2020), windows were convolved with a Gaussian of σ = 3 TR to smooth the transition between windows (Allen et al., 2014), and the window shifted with a step of 1 TR (Tu et al., 2019; Bonkhoff et al., 2020). The window cross-correlation produced 273 correlation matrices, representing the fluctuation of functional connectivity between the identified ICNs. These matrices are Fisher's Z transformed before being passed to step 3 for clustering analysis.

#### 2.5.3. Step 3: Clustering analysis for brain state estimation

Recurrent or repeating connectivity patterns in an fMRI scan are known as dynamic brain states. To identify these brain states clustering is performed using the k-means based clustering algorithm. The distance between clustering points was computed using the Manhattan distance (i.e., the “city-block”), which is the distance metric recommended for high-dimensional-space clustering (Aggarwal et al., 2001). The number of clusters is automatically computed by maximizing the ratio of within-cluster distance and between-cluster distance, and the optimal candidate is then manually estimated using the elbow method (Allen et al., 2014; Bonkhoff et al., 2020). For each subject the correlation matrices from step 2 were grouped into different clusters according to the distance from the clustering centroid. This results in state labels for each of the time windows which are used in the dynamic feature calculations in the next step in order to investigate the difference between the young and elderly adult groups.

#### 2.5.4. Step 4: Dynamic feature extraction

Next, the FC temporal characteristic evaluation as well as the dynamic graph analysis were performed. Following (Allen et al., 2014; Bonkhoff et al., 2020), using the state labels, four FC temporal characteristics were calculated as features for the between-group difference: (i) state fraction: the percentage of the total number of FC windows for one subject which take the given state; (ii) mean dwell times: the mean time a subject spent in a state without switching to another one; (iii) number of transitions: how many times a subject changed states; and (iv) transition probability matrix: the transition likelihood between the k connectivity states.

The rationale behind the dynamic graph analysis is that, with the FC potentially fluctuating with each time window, so too the topological structure of the graph can vary. For the dynamic graph analysis, as shown in step 4 in Figure 1, the ICNs were defined as the nodes in the graph and the FC between them as the edges, thus for each participant a graph is obtained for each time window. To define the adjacency of the nodes a threshold can be applied to the edges in the graph to produce an undirected and binary adjacency matrix. However, as the topological structure is not constant within one graph if using different network thresholds, the network sparsity method has been adopted in our experiment to avoid the bias of unstable measures in between-group dynamic feature comparison (Zhang et al., 2011; Kim et al., 2017; Xia et al., 2019; Rashid et al., 2021). Similar to prior studies (Hashmi et al., 2017; van den Heuvel et al., 2017; Tu et al., 2019), 10 thresholds ranging from 0.05 to 0.50 with a step of 0.05 were used to obtain the sparse network. Each threshold produced an adjacency matrix for each DFNC matrix.

Having obtained the adjacency matrices then, graph theory was applied to investigate the topological organization of the DFNC state and the series of graphs. Specifically, we use 12 graph metrics to measure the graph characteristics and dynamics during the fMRI scan. For example, network efficiency, measures how efficiently a node exchanges information or communicates with other nodes within a network. The other selected metrics include assortativity, global and local efficiency, and synchronization, which depict a brain function network's resilience, segregation, and integration. Detailed definitions of these graph metrics and their formulas are listed in Supplementary Table 1: Appendix 1. To balance the sparsity selection for the sequence of thresholds, the area under the curve (AUC) for the metric values was computed. Then, the AUC was utilized as a graph feature for further analysis.

#### 2.5.5. Step 5: Statistics and machine learning tests

The final step in the pipeline conducts statistical testing to examine the results. To obtain robust and reliable results on aging-related variations within and between groups, a non-parametric permutation test with 5,000 randomizations was implemented for all of the dynamic features produced in the DFNC analysis pipeline. The difference in the means of the distributions yielded after the 5,000 random permutations served as the t statistic. In addition, we investigated the presence of the distinct transient brain states across different age groups by performing a two-sample *t*-test. All statistical results were corrected by false discovery rate (FDR) for multiple comparison correction with a significance level of *p* < 0.05.

Meanwhile, nine machine learning algorithms were implemented to examine the power of the dynamic features to predict the age of an individual. These algorithms were exploited to learn a mapping from the raw fMRI space, X, to the age distribution of participants, Y. That is: Φ:G(X)→Y given the fMRI scan collection of training samples $T=\{\left(x_{n},y_{n}\right){\}}_{n=1}^{N}$. Here, *N* is the number of training sample scans, $x_{n}\in X$ is the input scan and $y_{n}\in Y$ is the associated age label indicating if the participant is an elderly adult. $G={\left\{g_{i}\right\}}_{i=1}^{V}$ is the function extracting dynamic FC features, and *V* is the number of features.

These algorithms were all implemented using the sklearn python package. For the 6 methods listed in Table 1 the default setup with the given parameters was used. In addition, we developed a neural network method using Keras's deep learning package. Considering our small sample data size could cause problems with over-fitting in the training phase for complicated network structures, a 2-layer forward neural network (FNN) was developed. The first and second layers of the neural network compose of 256 and 2 neurons (corresponding to the number of age categories). At the end of the first and second layers, there is a tanh and sigmoid activation function to learn the non-linear mapping relationship. The model is trained by minimizing the loss function:


(2) $$\begin{array}{l} L_{loss}=\frac{1}{N}\underset{i}{\sum }-\left[y_{i}\cdot \log\left(p_{i}\right)+\left(1-y_{i}\right)\log\left(1-p_{i}\right)\right], \end{array}$$

**Table 1:** Machine learning algorithms and their parameters.

**Algorithm**	**Parameters**
Nearest neighbors	Number of neighbors = 2
Linear SVM	Regularization parameter = 0.025
RBF SVM	Same as linear SVM
Gaussian process	Default
Decision tree	Depth = 5
Random forest	Number of neighbors = 5, number of estimators = 10

where *p*_*i*_ is the predicted probability. Finally, we test two ensemble fusion-based methods: one is Adaboost (Hastie et al., 2009), and the other one is Voting (Ruta and Gabrys, 2005). Both algorithms try to promote prediction performance by weighting multiple embedded estimators. In the Adaboost method, the default setup was opted for. In the voting method, the ensemble rule was set to be “hard”, which means that the predicted class labels for majority voting will be the final prediction results.

The dynamic feature output by *g*_*i*_ was singly fed into these machine learning methods to examine whether the aging group classification facilitates dynamic classification. In addition, we have also cascaded the outputs of G(X) together to examine if the concatenated dynamic feature can promote the performance.

## 3. Results

### 3.1. Intrinsic connectivity networks

**Figure 2:**
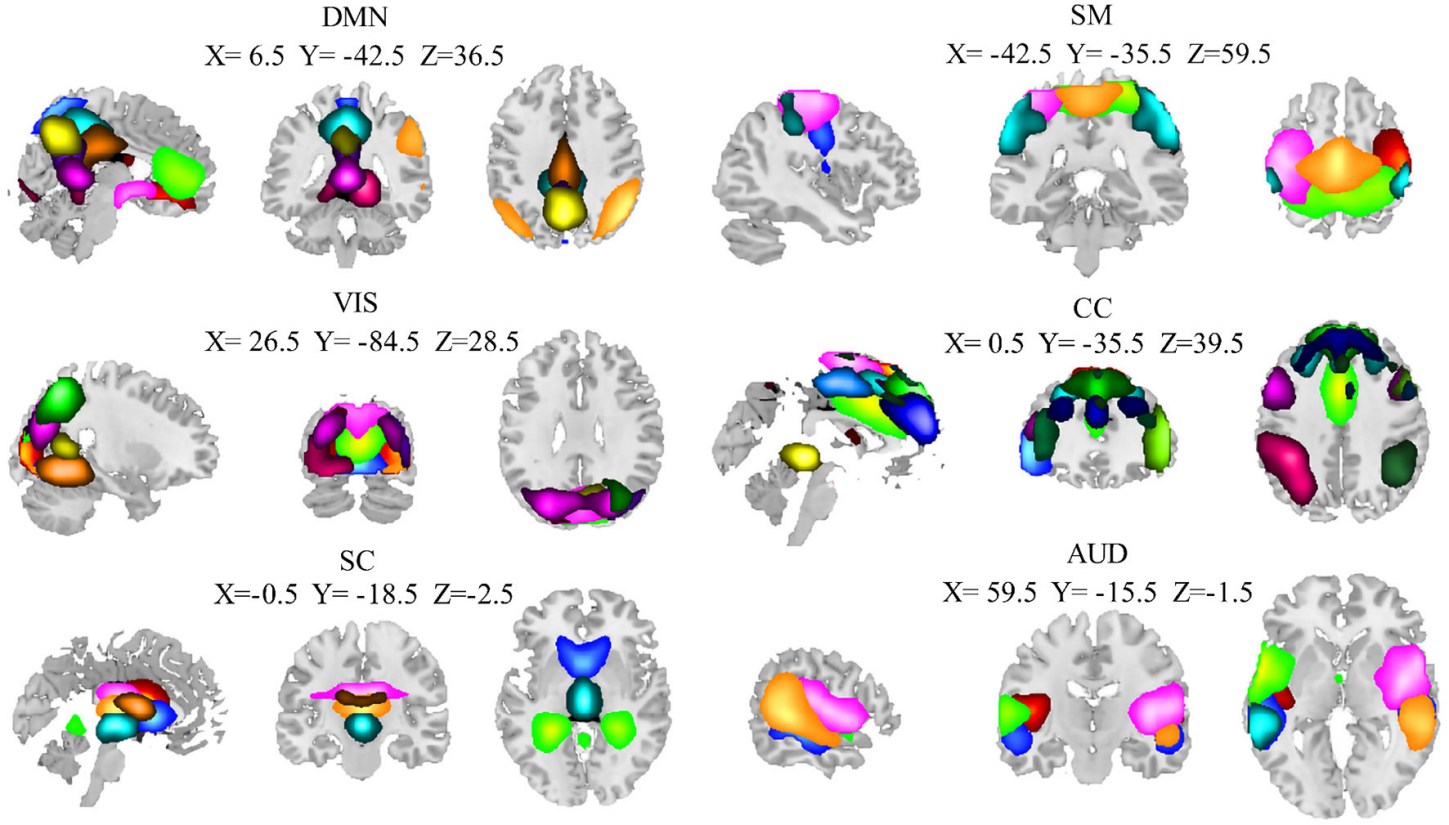
Spatial maps of the 60 independent components result from the entire group (28 elderly and 34 younger adults). The coordinates denote the max peak location of functional domains, and different colors pass spatial information. AUD, auditory domain; CC, cognitive control domain; DMN, default mode domain; SC, subcortical; SM, sensorimotor domain; VIS, visual domain.

Of the 100 ICs identified by the group ICA, 40 ICs were identified as noise components and then discarded. The remaining 60 components were finally identified as ICNs. The 60 ICNs were assigned to one of six domains that have been widely studied in normal aging (Xia et al., 2019; Snyder et al., 2021) (Figure 2): subcortical domain (SC), auditory domain (AUD), visual domain (VIS), sensorimotor domain (SM), cognitive control domain (CC), and default mode domain (DMN). The detailed component labels and peak coordinates of each ICN have been provided in the Supplementary material: Appendix 2.

### 3.2. Static functional network connectivity analysis

**Figure 3:**
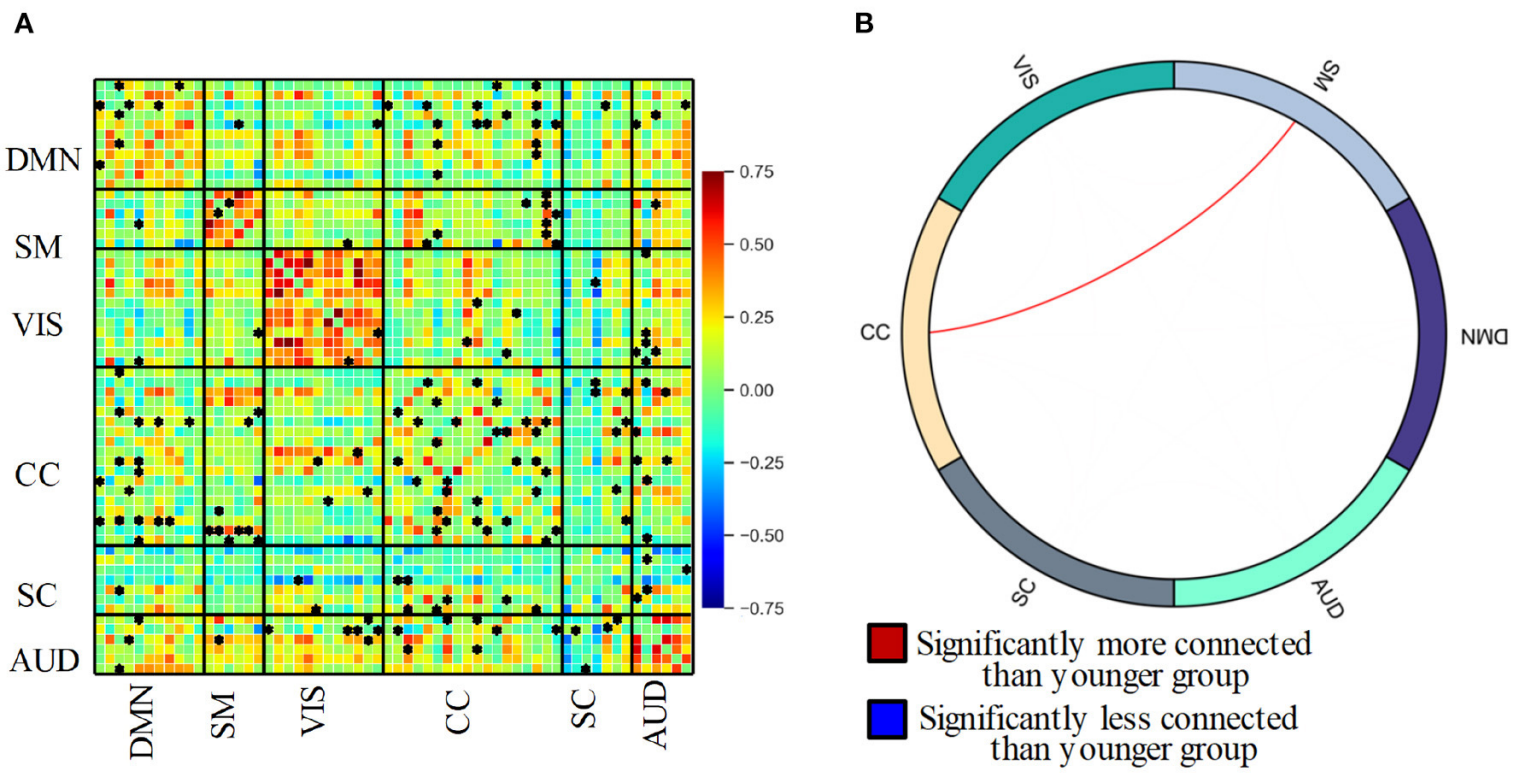
**(A)** Static functional network connectivity between 60 independent components resulting in 1770 ( 60 × (60-1)/2 )connectivity pairs for the entire group. Asterisks indicate significant differences between the elderly and younger groups. **(B)** Circle plot of significant static functional network connectivity differences of 6 domain between the elderly adult and younger group.

Figure 3 shows the static functional network connectivity aggregated over the entire scanning time series using the group ICA method. The red color indicates a positive correlation, and the blue color represents a negative correlation between functional spatial regions. With the static functional network connectivity, we observed strong intra-domain connectivity, i.e., connectivity within the DMN, SMN, VIS, and AUD domains was positively correlated. In contrast, the inter-domain connectivity was comparably low, where the functional regions in the 6 domains were either independent of each other or negatively connected. This phenomenon was particularly obvious for the SC domain, where the connectivity with the other 5 domains was nearly all negative. Within the SC domain, the brain areas also exhibit negative connectivity.

Further reviewing the difference in connectivity from the elderly group and the younger using a two-sample *t*-test, 193 connectivity pairs show significantly altered between-network connectivity components. The significant alterations in ICNs have been denoted with an asterisk in Figure 3A, from which we can see that these alterations are mainly related to the CC domain. Post *t*-tests, contrasting elderly adults and younger controls, reveals aging-induced reduced connectivity (*p* < 0.05, FDR-corrected). From Figure 3B, we can see only the connectivity between SM and CC domains was left after post *t*-tests in group ICA (*p* < 0.05, FDR-corrected). This result shows consistency with the studies that show higher connectivity between the somatosensory and control network (Geerligs et al., 2015).

### 3.3. Dynamic functional network connectivity analysis

#### 3.3.1. DFNC State

**Figure 4:**
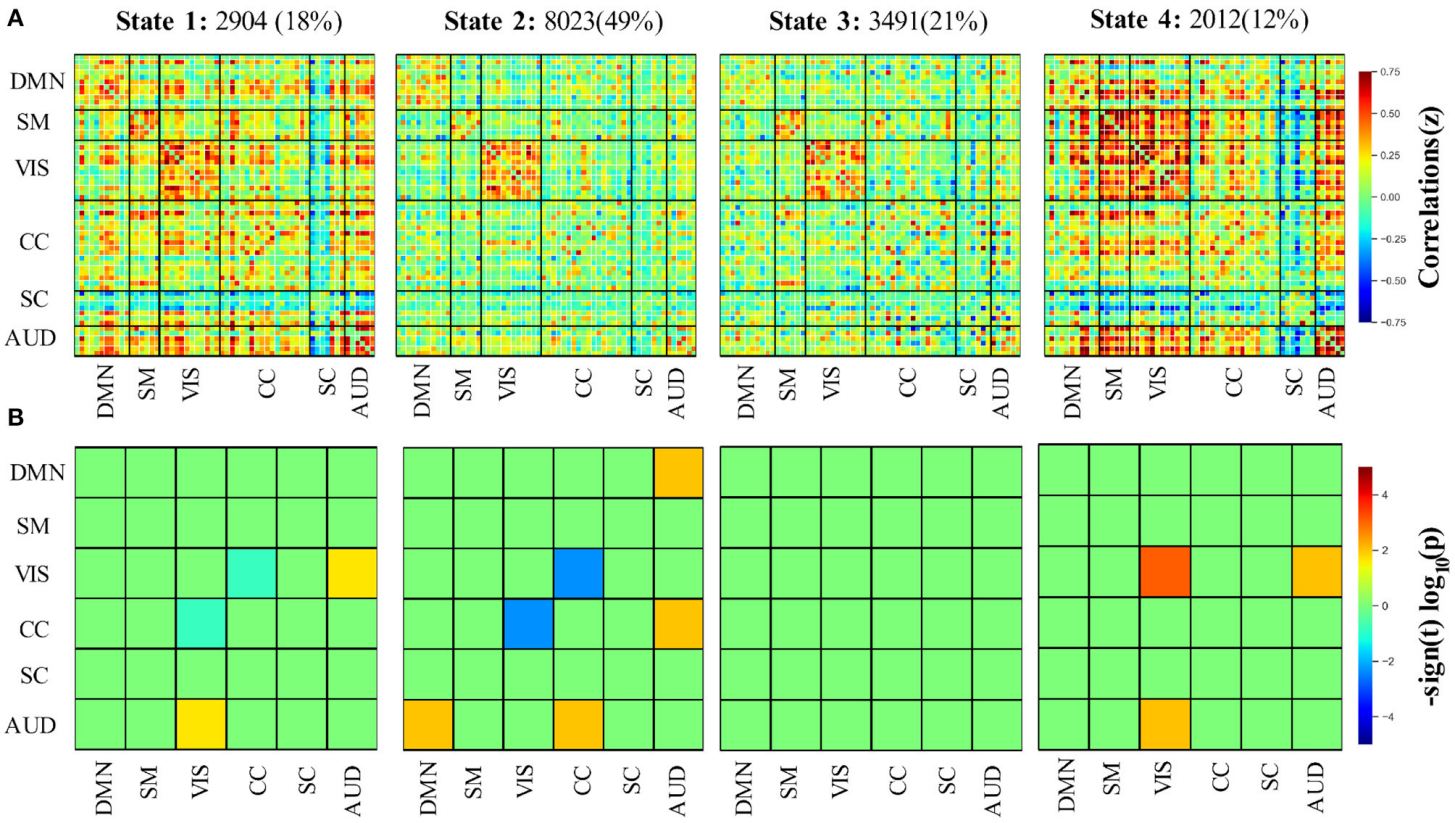
**(A)** Four functional connectivity states as well as their frequencies across all participants using the group-ICA method. **(B)** Group differences of the 6 selected brain networks between elderly and younger adults in the 4 states. AUD, auditory domain; CC, cognitive control domain; DM, default mode domain; SC, subcortical; SM, sensorimotor domain; VIS, visual domain.

Four DFNC states were identified from the clustering. The identified states were the functional patterns that frequently reoccurred across all the participants, and are stable characterizations of the brain activity during the fMRI scanning. The four states are presented in Figure 4A indexed with the order given by k-means.

According to the connectivity pattern, the states can be grouped into two categories. State 1 and 4 compose the first class, characterized by dense inter-and intra-domain connectivity. We can observe highly positive between-AUD domain connectivity and negative between-SC domain connectivity. State 1 closely matches the static connectivity in terms of Manhattan distance. The second category involves states 2 and 3. Compared with the first, this class featured relatively weak and sparse connectivity, which is particularly obvious for the SC and AUD domains. Thus, we refer to the category as the weakly connected class. The state frequency of two connectivity types also supports this classification, in which the frequencies of two states in class 1 are no more than 20%, which is less than that of class 2 (which accounts for 70% in total for all subjects). Meanwhile, it is worth noting that the strong positive connectivity within VIS can be observed for all 4 states.

Even though the DFNC states exhibit two categories, group differences for each state are varied (see Figure 4B). Within state 1, the elderly adults have slightly lower connectivity between VIS and CC while having relatively higher connectivity between VIS and the AUD domain (*p* < 0.05, FDR-corrected). In state 2, the connectivity between VIS and CC in the elder group shows a further decline. At the same time, significantly increased connectivity between DMN and AUD and CC and AUD can be found in this state. The only significantly different intra-domain connectivity was observed in state 4. The result shows that within state 4, the elderly group has markedly stronger connectivity in the VIS domain than younger adults (post *t*-tests: *p* < 0.05, FDR-corrected). Similar to state 1, the weaker connectivity between VIS and AUD domains can also be observed in state 4. We did not find any significantly different connectivity in state 3 between the two groups (post *t*-tests: *p* < 0.05, FDR-corrected). In contrast to the connectivity difference that the static connectivity state exhibits between the two groups, there is no significant difference between CC and SMN after the FDR-corrected in all 4 states.

#### 3.3.2. DFNC temporal features

**Figure 5:**
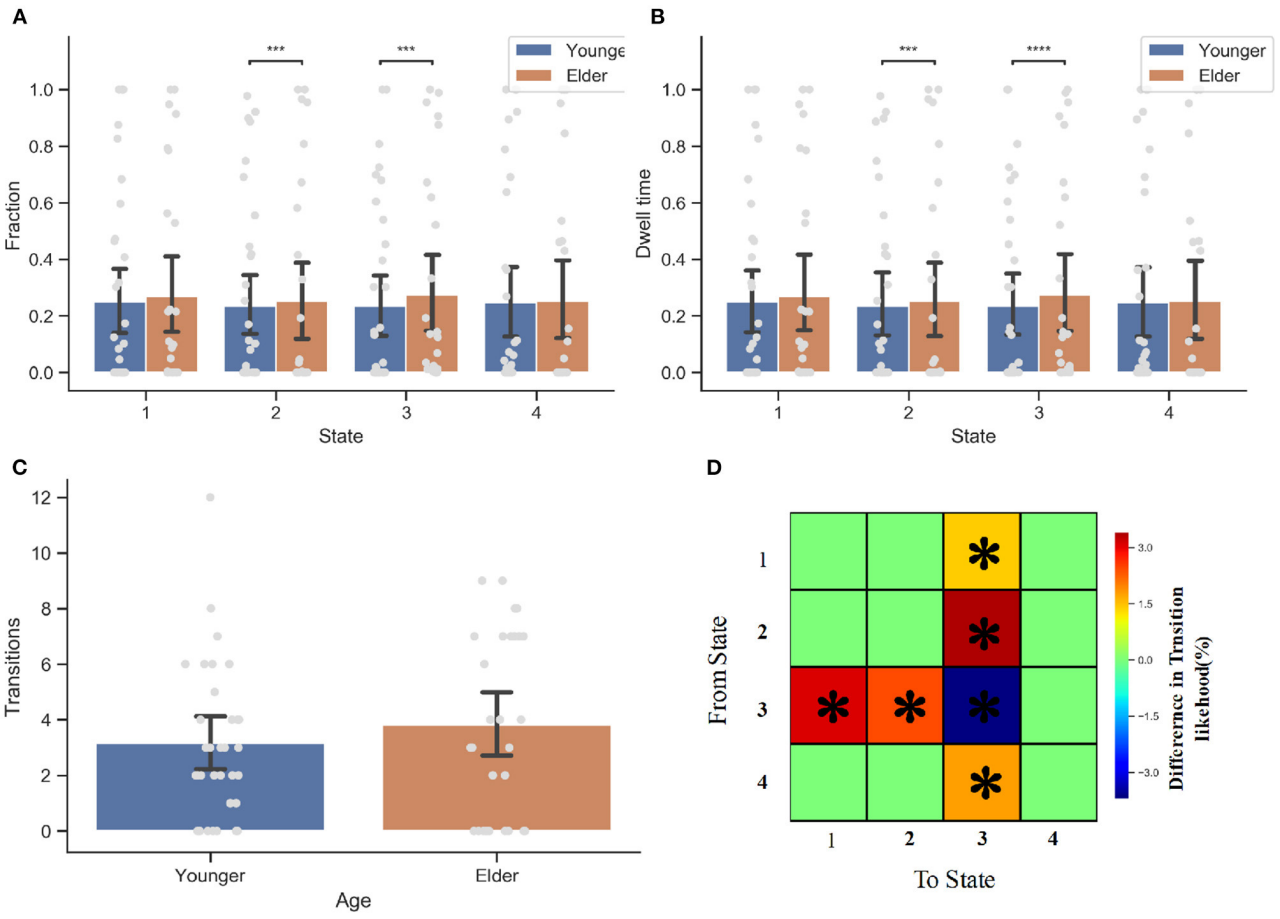
Dynamic connectivity feature analysis for the elderly and younger groups. **(A)** The fraction of time the occurrence of DFC state 2 and state 3 has significant between group difference. The elder prefer state 2 (*P* < 0.05) and state 3 (*P* < 0.05). **(B)** The dwell time. Once again, the senior group is more like to stay within state 2 and state 3. **(C)** The number of transition between states. There is no significant difference in the number of state transition between two groups. **(D)** State Transition Probability matrix. Comparing with younger adults, the older people more inclined to switch to state 3 when they are in state 1, 2, or 4. However, they are also more likely to transfer to other state when they are entering state 3 than younger people. *p < 0.05, ***p < 0.001, and ****p < 0.001 FDR corrected.

With four dynamic functional connectivity states and window-based FNC matrices, we subsequently tested for between-group differences in the measures of dynamic features (see Figure 5). Two sample *t*-tests comparing younger and elderly adults revealed a significant difference in the dynamic measures (fraction and dwell time) of state 2 as well as state 3 (i.e., the weak connectivity pattern, *p* < 0.05, FDR-corrected). In contrast to younger adults, the elderly prefer states 2 and 3 (*p* = 0.0001), and they are more likely to stay in states 2 and 3 once they enter these states (*p* = 0.0001). The between-group difference in dwell time of state 3 is more prominent (*p* < 0.0001). No significant between-group difference was observed in terms of the number of state transitions.

With respect to the transition probability matrix between states, there were significant between-group effects on the likelihood of staying in one state stably or switch to another. Consistent with the finding that the elderly prefer to spend more time on state 3, results showed that the elderly are more inclined than younger people to switch to state 3 when the current state is not state 3. This is particularly true when the current state is state 2 (*p* = 0.0001, FDR-corrected), demonstrating why the elderly prefer state 2 but have less dwell time than state 3. However, when entering State 3, older people are less likely to remain in this state than younger ones. More elderly people prefer to switch to state 1 or state 2, while younger people tend to maintain a stable state (*p* < 0.05, FDR-corrected). When the next state is state 4, the transition probabilities of elderly and younger people do not differ.

To better utilize the dynamic connectivity features to serve aging classification, we next explored the correlation between these features and the age of participants. The dynamic connectivity features correlated with age have been listed in Table 2. As can be seen, the fraction time and dwell time of state 2 are negatively correlated with age (fraction time: *r* = −0.639, *p* = 0.000; dwell time: *r* = −0.502, *p* = 0.000). In contrast, the fraction time and dwell time of state 3 are positively correlated with age (fraction time: *r* = 0.651, *p* = 0.000; dwell time: *r* = 0.555, *p* = 0.000). In terms of transition probability between states, the likelihood of state 1 switching to state 3, of state 3 switching to state 1, as well as state 3 switching to state 2 all have a positive correlation with age (*r* = 0.316, 0.265, 0.254, *p* = 0.012, 0.038, 0.046, respectively), while the probability of switching from state 3 to state 3 is negatively correlated with age (*r* = −0.409, *p* < 0.001).

**Table 2:** Dynamic state features correlated with age.

**Dynamic connectivity features**	***r***	***p***
Fraction time of state 2	−0.639	0.000
Fraction time of state 3	0.651	0.000
Dwell time of state 2	−0.502	0.000
Dwell time of state 3	0.555	0.000
Transition probability from state 1 to state 3	0.316	0.012
Transition probability from state 3 to state 1	0.265	0.038
Transition probability from state 3 to state 2	0.254	0.046
Transition probability from state 3 to state 3	−0.409	0.001

#### 3.3.3. Dynamic graph analysis

To explore the age effect on the functional network topology, the subsequent work employed graph theory to characterize the dynamic graph changing during fMRI scanning. Various graph metrics have been utilized, which can describe multiple network properties. These graph measures were calculated based on the sparsing-threshold binary networks per participant and then averaged within the group. Subsequently, they were tested for between-group differences in terms of graph dynamics.

**Figure 6:**
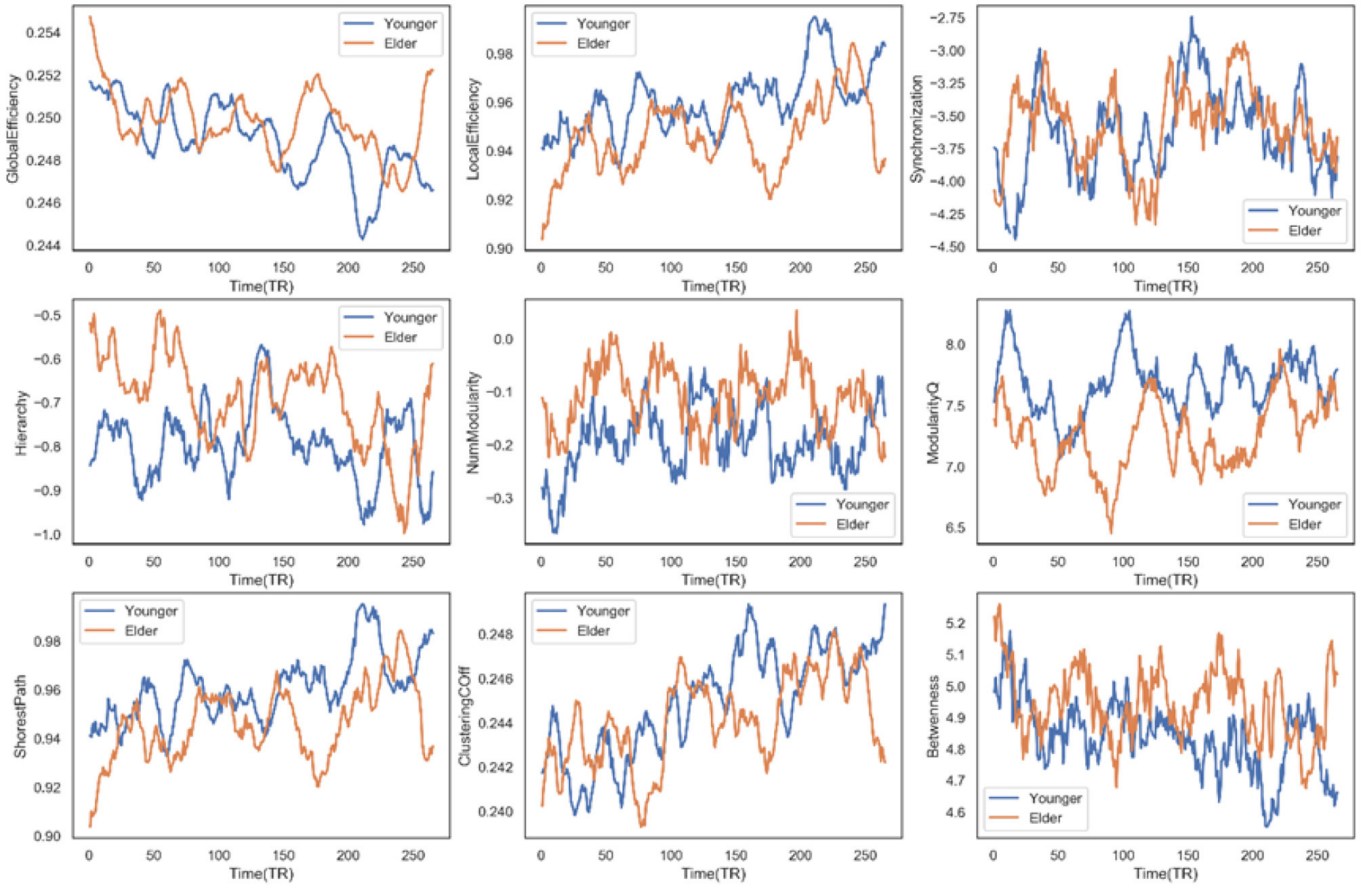
Time course of multiple dynamic measures for the different age groups.

Firstly, we observed a significant between-group difference in global efficiency (*t* = 6.5046, *p* < 0.0001), local efficiency (*t* = −11.4388, *p* < 0.0001), synchronization (*t* = 2.2756, *p* = 0.0232), hierarchy (*t* = 12.384, *p* < 0.0001), modularity (*t* = 16.1638, *p* < 0.0001), the shortest path (*t* = −11.4388, *p* < 0.0001), clustering coefficient (*t* = −4.1766, *p* < 0.0001), and the betweenness (*t* = 10.8943, *p* < 0.0001). Figure 6 displays the time course of these graph metrics. In terms of efficiency, we can see that the elderly group has a higher global but lower local efficiency than the younger group, suggesting that the information transfer is more efficient in the global but less efficient in the local functional network as age grows. Across these dynamic measures, the elderly people only have three measurements significantly higher than the younger group: the synchronization coefficient, hierarchy coefficient, and modularity. These higher measures indicate that as the age increases, the synchronization ability of the functional region in the brain network increases. The raised age increases modularity and enriches the hierarchy structure. Note that the significantly higher value in elderly people is not overwhelming. At some transient time points, these younger people have a stronger performance in these measures. Examples include the weaker synchronization in the younger group at TR = 150, which is consistent with the observed lower synchronization in transient dynamic state 2 for older adults. A significant difference can also be observed in the clustering coefficient and the shortest path, which directly results in the distinguishing small-world property of the two groups. The lower small-world property implies that the elderly group is less robust to external perturbations, according to the hypothesis by Barabási (2013). In that case, it fits our biological intuition that older people are subject to damage by mutation or viral infection. However, there may be a lack of direct evidence to demonstrate a linear relationship.

**Figure 7:**
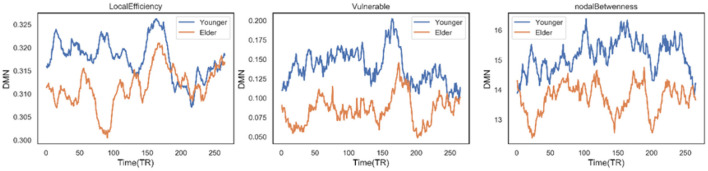
Time varying curve of the three dynamic measures of DMN network: local efficiency, vulnerable, nodal betweenness in age-different group, where no matter which metric elder people are lowest.

Secondly, there are three other nodal graph measures explored in our work: local efficiency, vulnerable coefficient, and betweenness, which characterize the information-transferring efficiency of a specific node, the vulnerability of a node, and the importance of the node's role in the network, respectively. Six sub-networks were observed to have significant differences between the two groups on the three measures. For the DMN network, people 60–80 years old have significantly lower local efficiency (*t* = −16.8892, *p* < 0.0001) and vulnerable coefficient (*t* = −31.5046, *p* < 0.0001) than younger people, suggesting the DMN has less efficient information transfer and a higher risk of slowing global efficiency. The same situation occurs in the VIS network (*t* = −19.9135 and −7.0166 for the two metrics, respectively). The DMN network efficiency decline can also be supported by the significantly decreased nodal betweenness (*t* = −31.8963, *p* < 0.0001), where the higher the nodal betweenness coefficient, the more likely information will transfer through the node. Figure 7 shows the time-varying curve of these three measures in DMN during the fMRI scan. We can see that older people's metric curves do not have an apparent trend, but they are always lower than younger people's. Besides, the correlation between the nodal graph measures and the age for the six domains also behaved differently (see Table 3). The DMN's local efficiency, vulnerable coefficient, and nodal betweenness have some of the highest negative correlation values compared to the other five networks. On the other hand, the CC domain has the smallest correlation in all three measures compared to other parts.

**Table 3:** The correlation between nodal graph measures and age.

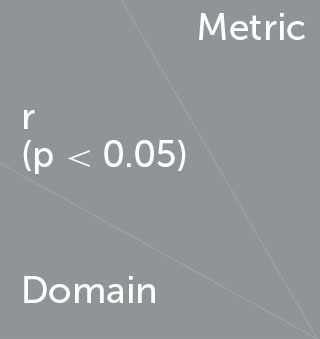	**Local efficiency**	**Vulnerable**	**Nodal betweenness**
DMN	−0.263	−0.433	−0.496
SMN	0.159	0.2121	0.364
VIS	−0.298	−0.117	0.237
CC	0.048	0.004	0.087
SC	0.252	0.137	−0.093
AUD	0.111	0.049	0.163

### 3.4. Machine learning test for individual age prediction

Given that the dynamic features are significantly different between the two groups, it is natural to test their power in individual age prediction using a machine learning algorithm.

Firstly, the single dynamic state feature (the fraction, MDT, etc.) was fed into the nine machine learning algorithms, respectively, to test their prediction power, with the static functional connectivity strength serving as baseline for comparison. The performance of each pipeline was evaluated with five-fold cross-validation, and the result of the test set is summarized in Table 4. The evaluation metric is accuracy, i.e., the probability that the method correctly categorizes the candidates into the correct class. We report the mean of five-fold cross-validation results in each metric with a 95% confidence interval.

**Table 4:** The prediction accuracy of multiple machine learning algorithms with dynamic state features.

		**Dynamic state feature**				
**Algorithm**	**Baseline**	**Fraction**	**MDT**	**Num of trans**.	**Transition probability**	**Concatenated**
Nearest neighbors	0.551(0.124)	*0.852 (0.117)*	0.636 (0.251)	0.690 (0.160)	0.648 (0.135)	0.795 (0.200)
Linear SVM	0.531 (0.112)	*0.852 (0.117)*	0.612 (0.152)	0.640 (0.107 )	0.648 (0.112)	0.840 (0.124)
RBF SVM	0.531 (0.112)	*0.852 (0.117)*	0.617 (0.207)	0.607 (0.119 )	0.631 (0.141)	0.740 (0.186)
Gaussian process	0.483 (0.106)	*0.838 (0.124)*	0.536 (0.170)	0.690 (0.141)	0.683 (0.1770)	0.729 (0.158)
Decision tree	0.585 (0.132)	***0.886** (0.129)*	0.583 (0.165)	0.729 (0.175)	0.648 (0.211)	0.840 (0.144)
Random forest	0.585 (0.153)	*0.855 (0.116)*	**0.650** (0.225)	0.755(0.157)	0.695 (0.175)	0.852 (0.157)
FNN	0.552 (0.173)	*0.838 (0.124)*	0.579 (0.155)	0.560 (0.118)	0.564 (0.104)	0.807 (0.161)
AdaBoost	0.577 (0.111)	0.807 (0.142)	0.617 (0.193)	**0.771** (0.150)	**0.731** (0.195)	***0.855** (0.138)*
Voting	0.511 (0.131)	*0.852 (0.117)*	0.617 (0.193)	0.624 (0.156)	0.679 (0.143)	0.840 (0.163)

The bold values represent the algorithm that achieves the best performance using the feature indicated in the column. The italic values denote the highest accuracy that the machine learning algorithm could obtain across all the input dynamic features.

As can be seen, by using the state fraction feature, all the machine learning algorithms have an accuracy over 80%, which is higher than any other feature (except the AdaBoost method with concatenated feature). The decision tree achieves the highest accuracy of 0.886 using this feature, which is also the best performance in all of the dynamic state features. The highest accuracy for the number of transitions and transition probability is similar, 0.771 and 0.731, respectively. Meanwhile, the best performance of the number of transitions is more stable than that of transition probability, where the accuracy variance is less by 0.04. However, the number of transitions has a large gap in performance in terms of the different methods, where it can only achieve an accuracy of 0.560 with the FNN algorithm. The MDT has the lowest accuracy of 0.650. Concerning the concatenated feature, even though the results are not much worse than those for the number of transitions and transition probability, the highest accuracy is only 0.855, which is still less than the state fraction. On the other hand, with respect to the classic methods, the FNN method is the most unstable one. It obtains a mean accuracy of 0.855 using fractions while it has a 0.560 when using the transition probability as input. For the two ensemble-fusion-strategy-based methods, the voting method did not perform well for individual age prediction, though its best result is still for the fraction feature. In contrast, the AdaBoost method has achieved the best performance three times, the most frequent optimal method.

Second, similar to the dynamic state features, the dynamic graph features were also input into different machine learning algorithms. However, the results were not impressive using the single graph features (see Tables 5, 6). All of the features do not achieve accuracy over 70%, the best accuracy was just 0.693 obtained by the SVM with the number of modularity.

**Table 5:** The prediction accuracy of multiple machine learning algorithms with dynamic graph features (I).

		**Dynamic graph feature**				
**Algorithm**	**Baseline**	**Global efficiency**	**Local efficiency**	**Synchronization**	**Hierarchy**	**Modularity Q**
Nearest neighbors	0.551 (0.124)	0.5262 (0.208)	0.505 (0.165)	0.481 (0.209)	*0.571* (0.183)	0.533 (0.129)
Linear SVM	0.531 (0.112)	0.555 (0.195)	0.517 (0.142)	0.502 (0.166)	0.506 (0.231)	0.564 (0.220)
RBF SVM	0.531 (0.112)	0.548 (0.066)	0.548 (0.656)	**0.548** (0.066)	0.548 (0.066)	0.548 (0.066)
Gaussian process	0.483 (0.106)	0.471 (0.271)	0.531 (0.154)	0.469 (0.217)	0.607 (0.141)	***0.617*** (0.111)
Decision tree	0.585 (0.132)	0.340 (0.155)	**0.567** (0.188)	0.407(0.172)	0.576 (0.172)	0.483 (0.168)
Random forest	0.585 (0.153)	0.483(0.121)	0.502 (0.157)	0.457 (0.150)	0.579 (0.172)	0.500 (0.226)
FNN	0.552 (0.173)	0.436 (0.161)	0.533 (0.129)	0.467 (0.211)	**0.648** (0.247)	0.483 (0.259)
AdaBoost	0.577 (0.111)	0.505 (0.188)	0.369 (0.228)	0.390 (0.186)	0.569 (0.302)	0.519 (0.209)
Voting	0.511 (0.131)	***0.676*** (0.150)	0.533 (0.217)	0.536 (0.139)	0.500 (0.206)	0.357 (0.129)

The bold values represent the algorithm that achieves the best performance using the feature indicated in the column. The italic values denote the highest accuracy that the machine learning algorithm could obtain across all the input dynamic graph features.

**Table 6:** The prediction accuracy of multiple machine learning algorithms with dynamic state features (II).

	**Dynamic graph feature**				
**Algorithm**	**NumModularity**	**ClusteringCOff**	**Shortest path**	**Betweenness**	**Concatenated**
Nearest neighbors	0.562 (0.200)	0.450 (0.171)	0.531 (0.186)	0.529 (0.194)	0.437 (0.049)
Linear SVM	***0.693*** (0.133)	0.486 (0.164)	**0.564** (0.165)	0.500 (0.032)	0.515 (0.074)
RBF SVM	0.548 (0.066)	0.548 (0.066)	0.548 (0.066)	0.548 (0.066)	0.548 (0.019)
Gaussian process	0.598 (0.123)	0.448 (0.122)	0.500 (0.152)	**0.598** (0.105)	0.548 (0.019)
Decision tree	*0.579* (0.201)	0.436 (0.143)	0.419 (0.182)	0.567 (0.115)	**0.610** (0.153)
Random forest	0.517 (0.168)	***0.581*** (0.162)	0.467 (0.189)	0.579 (0.155)	0.421 (0.197)
FNN	*0.662* (0.082)	0.367 (0.149)	0.514 (0.198)	0.550 (0.143)	0.533 (0.012)
AdaBoost	*0.650* (0.121)	0.310 (0.141)	0.507 (0.152)	0.581 (0.207)	0.579 (0.068)
Voting	0.511 (0.222)	0.474 (0.208)	0.529 (0.099)	0.593 (0.188)	0.529 (0.099)

The bold values represent the algorithm that achieves the best performance using the feature indicated in the column. The italic values denote the highest accuracy that the machine learning algorithm could obtain across all the input dynamic graph features.

## 4. Discussion

Given the known dynamic nature of brain activity, it is reasonable to use the DFNC method to investigate the differences in dynamics between age groups. In the study presented here, four transient brain states that frequently reoccur at rest were identified. These 4 states exhibit two types of connectivity patterns: the densely inter-and intra-domain connectivity pattern and the weakly sparse one. The elderly tend to transfer to and stay in the weakly connected state, which cannot be shown with static analysis. Notably, the fraction of these DFNC states and the dwell time were correlated with age (*r* = 0.6392/0.6507 for time fraction of state 2 and 3, respectively; *r* = 0.5022/0.5553 for the dwell time of state 2 and 3, respectively). Besides, these dynamic measures gain advantage in brain age classification compare to static ones. The fraction time of DFNC state can achieve highest accuracy of 0.8857 using a decision tree.

There is a significant difference in the dynamic graph topology found between the young group and the elderly group. Older people have higher global but lower local information transformation efficiency, stronger synchronization ability, increased betweenness, more rich modularity and hierarchy structure, shorter shortest path length, and a declining clustering coefficient than younger people. At the nodal level, elderly adults differed from younger people in terms of local efficiency, vulnerable coefficient, and betweenness. The most notable of these differences is that the information transfer efficiency, the vulnerability, and the nodal betweenness of older people's DMN are all less than those of the younger group during the rest period. Thus, we here substantiated the lower role of DMN in elderly people, indicating dynamic analysis's benefit.

### 4.1. The correlation between dynamic features and mnemonic discrimination ability

Mnemonic discrimination ability (MDA) is the perception ability of humans to distinguish existing memories from current inputs by retrieving and encoding past events or experiences. Studies have shown that the decrease in MDA is a sign of neurodegenerative diseases relative to aging. Many pieces of evidence show that as age increases, the MDA will significantly decline (Stark et al., 2013, 2019; Wahlheim et al., 2022). However, whether the relationship is linear or not is not clear.

MDA is usually measured by the lure discrimination index (LDI), calculated as the difference in similar responses to lures and foils in the mnemonic discrimination task (Stark et al., 2019). Previous studies have demonstrated the DMN network has an age-inducted abnormal connectivity (Raichle, 2015; Nash et al., 2021), and this connectivity abnormality can develop a positive prediction model for LDI (Wahlheim et al., 2022). Nevertheless, this prediction is based on the static connectivity strength, the dynamic characteristics of DMN, or broadly, the function sub-networks, have not been thoroughly investigated. Hence, with the LDI provided by the original data source, this section additionally investigates the correlation between age, the dynamic feature, and MDA.

Firstly, age was observed to be negatively correlated with LDI (*r* = −0.3890, *p* = 0.001), which is consistent with the previous findings (Reagh et al., 2016). In terms of dynamic state features, the fraction time of state 2 is positively correlated with LDI (*r* = 0.3270, *p* = 0.0094), and the fraction time of state 3 is negatively correlated with LDI(*r* = −0.3882, *p* = 0.0018). Similar to fraction time, the MDT of states 2 and 3 has a significant correlation with LDI, where the correlation is *r* = 0.3145 (*p* = 0.0127) and *r* = −0.3591 (*p* = 0.0041), respectively. There is no significant correlation between the number of transitions and LDI or between transition probability and LDI. Recall that the connectivity pattern of state 3 is both weakly connected. This finding implies that the transient weakly connected state impacts the ability of everyday people to distinguish objects. We speculate the aging brain regulates the fraction of the weak state and its dwell time to determine the perceptive ability. In the weak state, the ability of different brain regions to communicate and coordinate with one another is reduced. As age increases, the brain cannot afford the active connectivity state and prefer a “standby” or “sleep” mode, thus lowering the perceptive function. In addition, cognitive and perceptual changes may be interrelated since they are both susceptible to age-related factors, meaning that a reduction in the functioning of the perceptual system may have an impact on cognitive abilities. Hence, it is possible to speculate that the common finding of cognitive decline in the aging brain could be closely related to the weak state of the brain. However, further experiments are necessary to confirm these speculations and explore the relationship between DFNC differences and health and cognitive function during aging. In addition, compared with state 3, state 2 has obvious positive connectivity within the DMN network, especially between the right angular gyrus and the anterior cingulum, suggesting that the transient state with positive connectivity in the DMN domain may promote the increase of MDA. In fact, previous studies have reported that connections positively related to mnemonic discrimination are broadly distributed across prefrontal, temporal, and parietal regions (Huijbers et al., 2011; Sestieri et al., 2011; Kim, 2016). Thus, we subsequently investigate the correlation between the nodal-level graph measure of DMN and LDI to hopefully extend our understanding of the DMN network's role in MDA.

The results show that only the node betweenness of DMN was observed with a weakly positive correlation (*r* = 0.2644, *p* = 0.03780) among the three nodal graph measures. According to the definition of nodal betweenness, this finding implies that the more information transfer passes through the DMN functional region, the more MDA. Besides, recall the highly negative relationship between DMN nodal betweenness and age. One possible and reasonable reason for the older adults' MDA being significantly lower than younger ones is that the aging process mitigates DMN participation gradually, thus inducing the decrease in the MDA. However, it may involve a complicated process. To substantiate this implication, more detailed experiments that target the brain DMN function domain are needed.

### 4.2. Dynamic balance of functional integration and segregation in healthy aging

The brain system keeps normal functions by maintaining the balance of functional integration (of different functional regions' information transmission for function response) and segregation (specialized information processing within the isolated functional regions). In many diseases with psychiatric disorders like schizophrenia, the disrupted balance between segregation and integration within the brain functional network has been demonstrated (Wang et al., 2016; Duan et al., 2019). Previous studies in human aging also revealed the abnormal integration and segregation within the brain function system: the decreased segregation occurs in the healthy aging process (Chan et al., 2014; Wig, 2017). Usually, the balance between integration and segregation can be quantified with small-worldness, a graph measure based on the trade-off between high local clustering and short path length (Humphries and Gurney, 2008). This network-level metric measures a graph with many local connections and a few random distance connections. Below, we calculate the dynamic small-worldness to investigate the time-varying balance of integration and segregation.

Firstly, the 2-way ANVOA result shows that age has no significant effects on the small-worldness measure (*F* = 2.18, *p* = 0.14), even though this measure is different between transient states. It suggests that the small-world network has not functionally changed as one ages. From the time-varying curve of small-worldness across the entire rest period, we can see that the small-worldness of both young people and the elderly has no clear boundaries. Most of the time, two curves are interwoven together. No one is always higher or lower than another. Besides, the two small-worldness curves are not smooth during the entire rest period. They have large fluctuations, with many spikes. What the spikes mean for the people's behaviors or if their characteristics, like the number of spikes and the energy, cause the age difference has not been clear. However, the measured value always fluctuates around 1 as time goes by, which means that both younger and older people keep a dynamic balance of functional integration and segregation.

Subsequently, from other graph metrics, we may have some clues to the changed functional integration and segregation in elderly people. As a spatially isolated functional specialization, segregation has multiple ways to be quantified. For example, previous studies have quantified segregation with the relationship connectivity strength within and between the modules (Chan et al., 2014; Wig, 2017; Bonkhoff et al., 2020). Hence, segregation is often connected with brain modularity. The higher the value of modularity, the more segregation in functional domains. Recall the modularity measure curves in Figure 6. The elderly's modularity is nearly always lower than the young, which perhaps implies more functional segregation in senior group people. However, a prior study in a long-term observation has demonstrated that the modularity and segregation might follow a U-shaped curve (Duncan and Small, 2016). Thus, the simple linear relationship between modularity and segregation in terms of age may not be true, and more evidence is needed to support that.

## 5. Conclusion

Aging has a profound influence on brain functional connectivity. This paper employed the DFNC method to explore the altered dynamic brain function interaction using the resting fMRI scans. Compared with static approach, the DNFC can capture the transient brain state in the elderly as well as young adults. The statistical analysis shows that the state-related features are significantly different between senior adults aged 60–80 and younger adults aged 18–30. In addition, DFNC exhibits the graph topology change spanning the entire scan, suggesting that growing age will induce an alteration in the information transformation efficiency, the robustness of the brain function network, and the dynamic balance of brain integration and segregation. Furthermore, this paper demonstrates that the time fraction of a transient stage could assist in brain age prediction due to the essential clues it carries (with the highest accuracy of 0.88). Overall, using a DFNC approach allows new insights into the systems-level effects that brain aging has on dynamic neural interaction, highlighting that the human brain tends to form differential function coupling patterns with aging. In future work, this function pattern alteration would be promising to help us interpret the relationship between aging and elderly-related diseases such as Alzheimer's disease or stroke.

## Data availability statement

Publicly available datasets were analyzed in this study. This data can be found here: https://openneuro.org/datasets/ds003871/versions/1.0.2. The jupyter notebooks for statistical evaluations and visualizations of this paper can be found here: https://github.com/xiaohajiayouo/Tracking_Functional_Network_Connectivity_Dynamics_in_the_Elderly.

## Ethics statement

The studies involving human participants were reviewed and approved by the Ethics Committee of the First Affiliated Hospital of Shantou University Medical College. Written informed consent for participation was not required for this study in accordance with the national legislation and the institutional requirements.

## Author contributions

KW, BJ, KN, and JF contributed to the conception and design of the study. KW organized the database and wrote the first draft of the manuscript. All authors contributed to manuscript revision, read, and approved the submitted version.

## Footnotes

^1^ https://openneuro.org/datasets/ds003871/versions/1.0.2
