# Supplementary material for: Tracking functional network connectivity dynamics in the elderly
Source: Front Neurosci. 2023 Mar 20;17:1146264. doi: 10.3389/fnins.2023.1146264 (PMC10069653; doi:10.3389/fnins.2023.1146264)
Supplement: Supplementary file 1 [file Data_Sheet_1.PDF]

Supplementary Materials for "**Tracking Functional Network Connectivity Dynamics in the Elderly**"

Appendix 1: Introduction to graph metrics

Let ICNs be the nodes and their pairwise connection be the edge, there will be graphs showing the dynamic topological alteration during the rest fMRI scan. Given the graph is $G = (V,E)$ which consists of a set of vertices V and a set of edges E, the number of nodes $n = |V|$ and the number of edges $e = |E|$. Then, the graph can be represented by an adjacency matrix $A \in \mathbb{R}^{n\times n}$ whose (i,j)-entry is defined as $A_{ij} = \{ {}^{1\ if\ ij \in E}_{0\ if\ ij \notin E} .$

Table I. Introduction to graph metrics

Metrics	Definition	Calculation
Graph level		
Assortativity (Newman, 2002)	The correlation coefficient between the degrees of pairs of linked nodes. Assortativity quantifies the tendency of nodes being connected to similar nodes in a complex network	$AC = \frac{e^{-1}\sum_{(i,j)\in E} k_i k_j - \left[e^{-1}\sum_{(i,j)\in E}(k_i + k_j)\right]^2}{e^{-1}\sum_{(i,j)\in E}(k_i^2 + k_j^2) - \left[e^{-1}\sum_{(i,j)\in E}(k_i + k_j)\right]^2}$ where k_i is the degree of node i.
Global efficiency	A measure of how efficiently a graph exchanges information.	$E_{global} = \frac{1}{n}\sum_{i\in V} E_{loci} = \frac{1}{n}\sum_{i\in V}\frac{\sum_{j\in V, j\neq i} d_{ij}^{-1}}{n-1}$ d_{ij} is the length of a shortest path between node i and j
Clustering coefficient	A measure quantifies how close the neighbors of nodes clustering together in a graph	$Clu = \frac{1}{n}\sum_{i\in N} C_i = \frac{1}{n}\sum_{i\in N}\frac{\sum_{j,h\in N} A_{ij}A_{ih}A_{jh}}{k_i(k_i - 1)}$
Synchronization (Arenas, 2008)	Measuring the synchronizability of a graph. Smaller the synchronization coefficient, the more synchronizable the network, and vice versa.	$Syn = \frac{\lambda_N}{\lambda_2}$ Where λ_i is the i-*th* eigenvalue of A.
Hierarchy	Measures the degree of hierarchy structure.	$\beta = reg(log(C), log(K), 'linear', 'beta')$ Where reg is function calculate the regression coefficient between nodal degree K and clustering coefficient C
Modularity (Newman, 2006)	A measure of the structure of a graph, measuring the density of connections within a module or community	$Q = \sum_{u\in M}\left[l_{uu} - \left(\sum_{v\in M} l_{uv}\right)^2\right]$ Where the network is divided into a set of nonoverlapping modularity M and l_{uv} is ration of all links that connect nodes in modularity u with module v
Shortest path length	The average number of steps along the shortest paths for all possible pairs of nodes in a graph.	$Spl = \frac{1}{n\cdot(n-1)}\sum_{h\neq j}\rho_{hj}$ Where ρ_{hj} is the number of shortest paths between h and j.
Nodal level		

Local efficiency	A measure of how efficiently a node exchanges information.	$E_{loci} = \sum_{i \in V} \frac{\sum_{j \in V, j \neq i} d_{ij}^{-1}}{n-1}$
Betweenness (Barthelemy, 2005)	A node with higher betweenness centrality would have more control over the network, because more information will pass through that node.	$B = \sum_{i \in V} B_i = \sum_{i \in V} \frac{1}{(n-1)(n-2)} \sum_{h,j \in V,\ h \neq j, h \neq i, i \neq j} \frac{\rho_{hj}^{i}}{\rho_{hj}}$ Where ρ_{hj}^{i} the number of shortest paths between h and j that pass through i.
Vulnerability	Vulnerability measures the susceptibility of the graph to fragmentation.	$Vuln_i = \sum_{h,j \in V,\ h \neq j, i \neq i, i \neq h} \frac{\rho_{hj}^{\bar{i}}}{\rho_{hj}}$ Where $\rho_{hj}^{\bar{i}}$ the number of shortest paths between h and j after removing node i.

Reference

Newman, Mark EJ. "Assortative mixing in networks." *Physical review letters* 89.20 (2002): 208701.

Arenas, Alex, et al. "Synchronization in complex networks." *Physics reports* 469.3 (2008): 93-153.

Newman, Mark EJ. "Modularity and community structure in networks." *Proceedings of the national academy of sciences* 103.23 (2006): 8577-8582.

Barthelemy, Marc. "Betweenness centrality in large complex networks." *The European physical journal B* 38.2 (2004): 163-168.

Appendix 2: Peak coordinates of ICNs

Table I. Peak coordinates of ICNs

ICNs	X	Y	Z
**Default-mode Domain: 11**			
Anterior cingulum cortex (18)	-0.5	25.5	-13.5
Precuneus (19)	-0.5	58.5	64.5
Anterior cingulum cortex (31)	-0.5	-34.5	4.5
Anterior cingulum cortex (52)	-0.5	-4.5	-10.5
Angular gyrus (53)	47.5	-64.5	37.5
Post cingulum + Precuneus (55)	-0.5	-43.5	43.5
Post cingulum (64)	-0.5	-28.5	26.5
Post cingulum (74)	14.5	-46.5	-1.5
Post cingulum + Precuneus (86)	-0.5	-62.5	19.5
Post cingulum + Precuneus (87)	-0.5	-64.5	37.5
Post cingulum (94)	-0.5	-46.5	4.5
**Sensorimotor Domain: 6**			
Right precentral gyrus (1)	35.5	-19.5	67.5
Postcentral gyrus (15)	-57.5	-7.5	25.5
Superior parietal lobule (20)	23.5	-43.5	67.5
Left precentral gyrus (21)	-36.5	-22.5	61.5
Paracentral lobule (25)	-0.5	-22.5	58.5
Postcentral gyrus (48)	59.5	-25.5	34.5
**Visual Domain: 12**			
Middle occipital gyrus (23)	29.5	-91.5	1.5
Lingual gyrus (17)	-0.5	-82.5	-7.5
Cuneus (30)	2.5	-85.5	10.5
Cuneus (34)	2.5	-76.5	43.5
Middle temporal gyrus (34)	47.5	-64.5	-19.5
Lingual gyrus (37)	-0.5	-64.5	-4.5
Lingual gyrus (47)	-23.5	-64.5	-13.5
Left Lingual gyrus (57)	-24.5	-76.5	-16.5
Middle temporal gyrus (58)	53.5	-67.5	1.5
Calcarine (67)	14.5	-61.5	7.5
Cuneus (79)	32.5	-76.5	22.5
Cuneus + precuneus (89)	26.5	-64.5	49.5
**Cognitive-control Domain: 18**			
Supplementary motor area (23)	-0.5	-28.5	-58.5
Left inferior frontal gyrus (28)	-51.5	34.5	-1.5
Middle cingulum (33)	-0.5	10.5	-34.5
Supplementary motor area (35)	-0.5	-4.5	64.5
Left Insula (39)	-36.5	7.5	-25.5
Superior frontal gyrus (42)	-24.5	19.5	49.5
Insula (43)	-42.5	13.5	-7.5
Left Inferior parietal lobule (46)	-39.5	-43.5	43.5
Left Lingual gyrus (60)	-12.5	-52.5	-1.5
Post cingulum (63)	-3.5	-37.5	-7.5
Middle frontal gyrus (65)	-51.5	13.5	28.5
Superior medial frontal gyrus (68)	-0.5	46.5	43.5
Insula (70)	-42,5	-7.5	-13.5
Right inferior frontal gyrus (73)	56.5	16.5	19.5
Middle frontal gyrus (78)	-33.5	55.5	-1.5
Middle frontal gyrus (83)	-0.5	52.5	19.5
Supplementary motor area (85)	-0.5	-16.5	49.5
Right Inferior parietal lobule (88)	44.5	-46.5	52.5
**Sub-cortical Domain: 7**			
Caudate (25)	-0.5	-4.5	16.5
Caudate (40)	-0.5	7.5	1.5
Putamen (49)	-27.5	-46.5	-1.5

Caudate (99)	5.5	-1.5	10.5
Caudate (66)	8.5	-19.5	19.5
Posterior thalamus (76)	-0.5	-22.5	7.5
Medial thalamus (90)	-0.5	-22.5	-4.5
**Auditory Domain: 6**			
Superior temporal gyrus (69)	-54.5	-25.5	10.5
Superior temporal gyrus (75)	-57.5	-37.5	-4.5
Superior temporal gyrus (77)	-60.5	-1.5	-1.5
Right Superior temporal gyrus (91)	44.5	-7.5	-1.5
Right Superior temporal gyrus (96)	59.5	-43.5	4.5
Left Superior temporal gyrus (97)	-54.5	-58.5	16.5

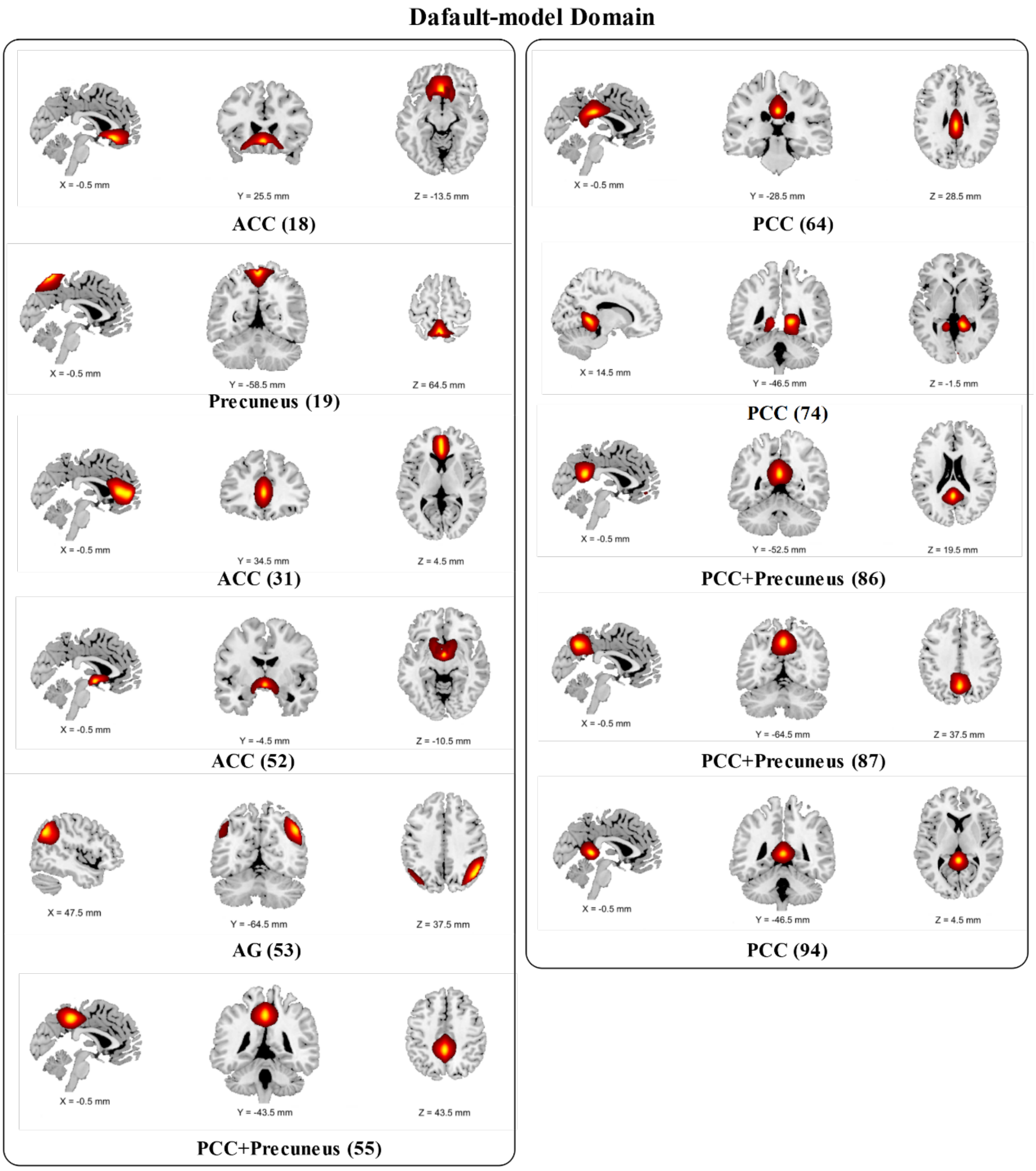

Figure 1-1. Spatial maps of intrinsic connectivity networks in default mode domain. Sagittal, coronal, and axial slices are shown at the maximal t-statistic for clusters larger than 3 cm^3.

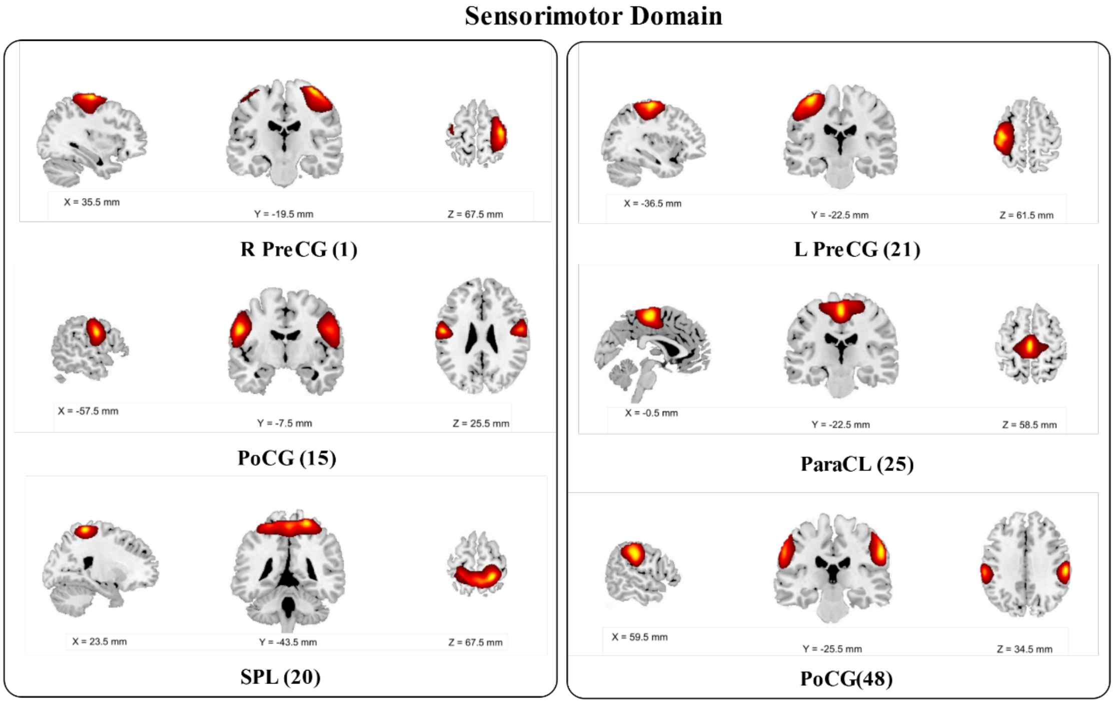

Figure 1-2. Spatial maps of intrinsic connectivity networks in sensorimotor domain. Sagittal, coronal, and axial slices are shown at the maximal t-statistic for clusters larger than 3 cm^3.

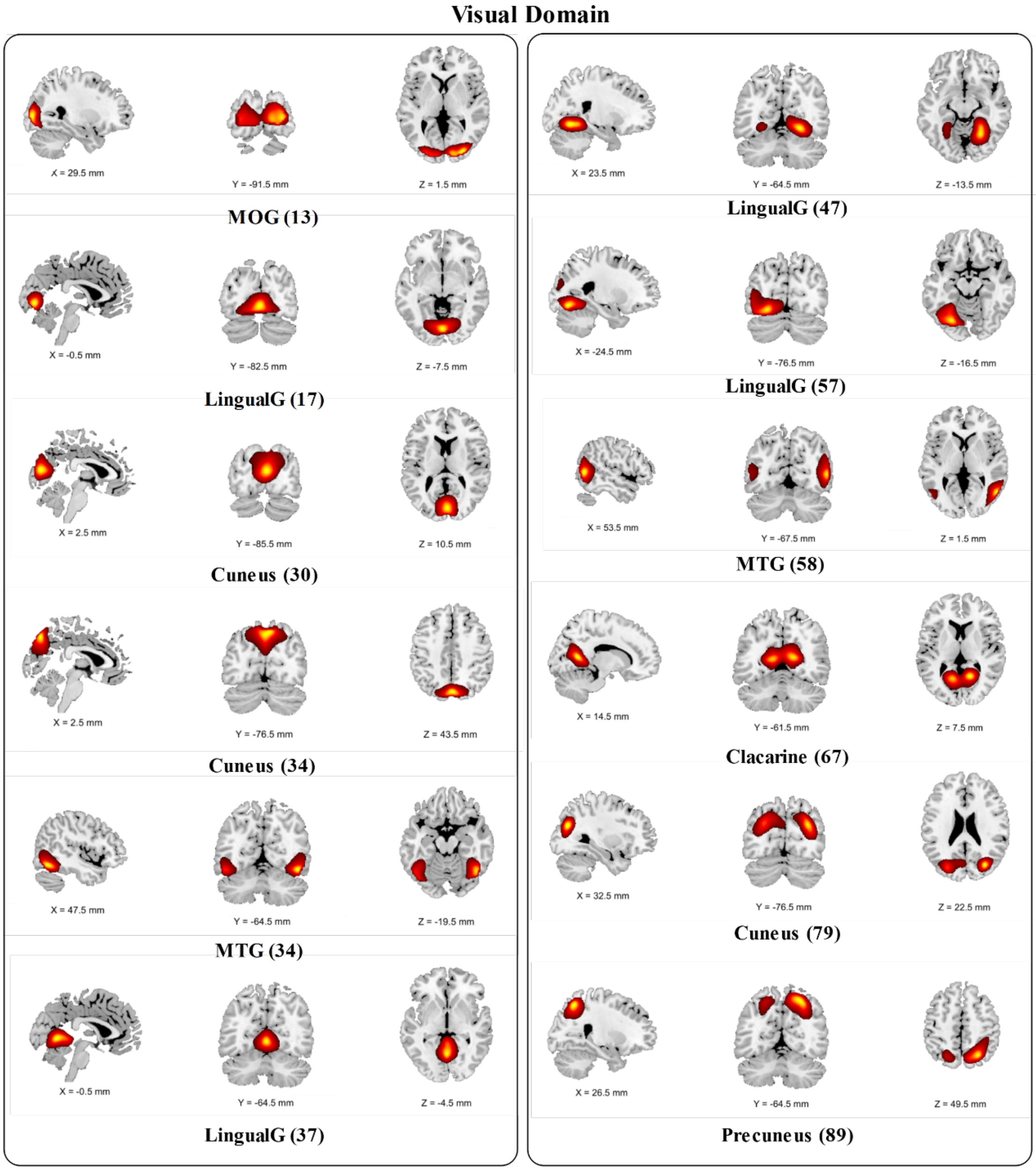

Figure 1-3. Spatial maps of intrinsic connectivity networks in visual domain. Sagittal, coronal, and axial slices are shown at the maximal t-statistic for clusters larger than 3 cm^3.

Cognitive-control Domain(1)

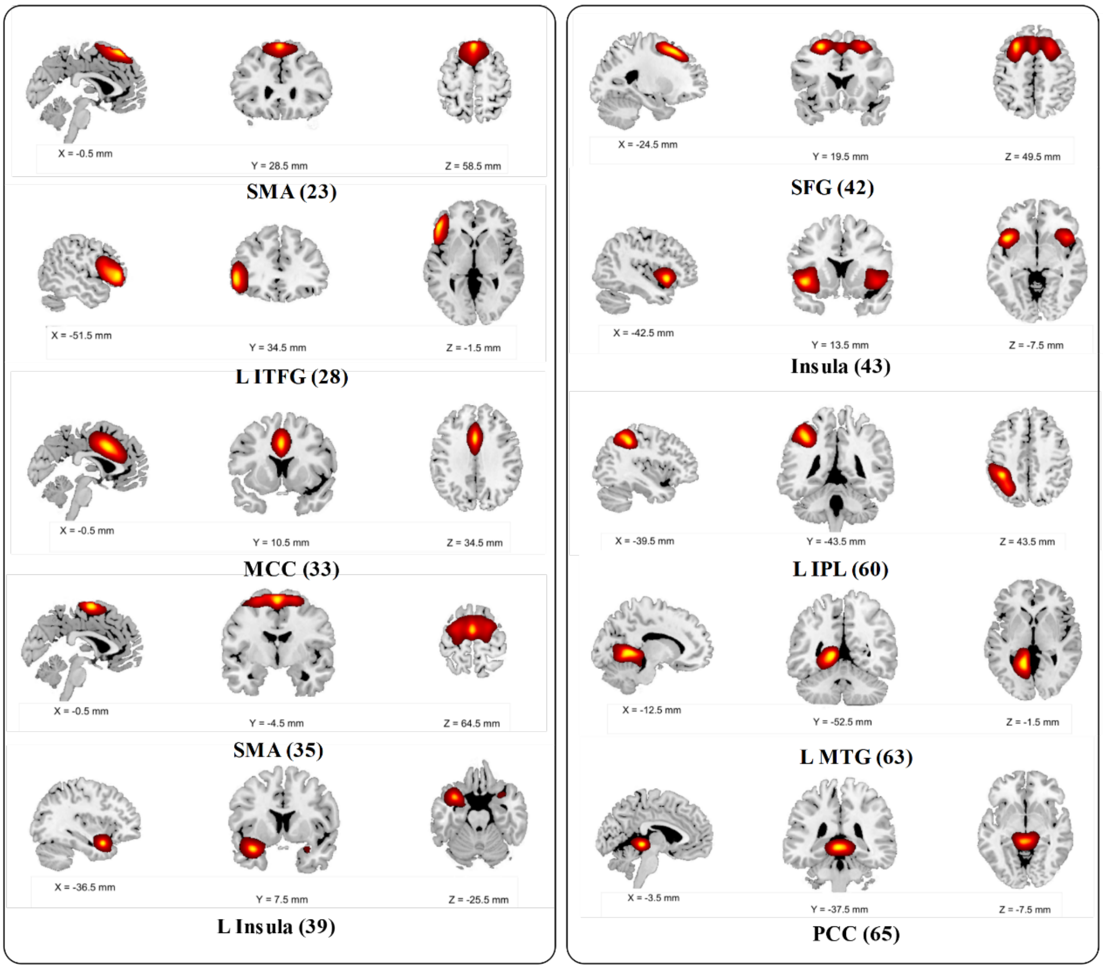

Figure 1-4(1). Spatial maps of intrinsic connectivity networks in cognitive control domain. Sagittal, coronal, and axial slices are shown at the maximal t-statistic for clusters larger than 3 cm^3.

Cognitive-control Domain(2)

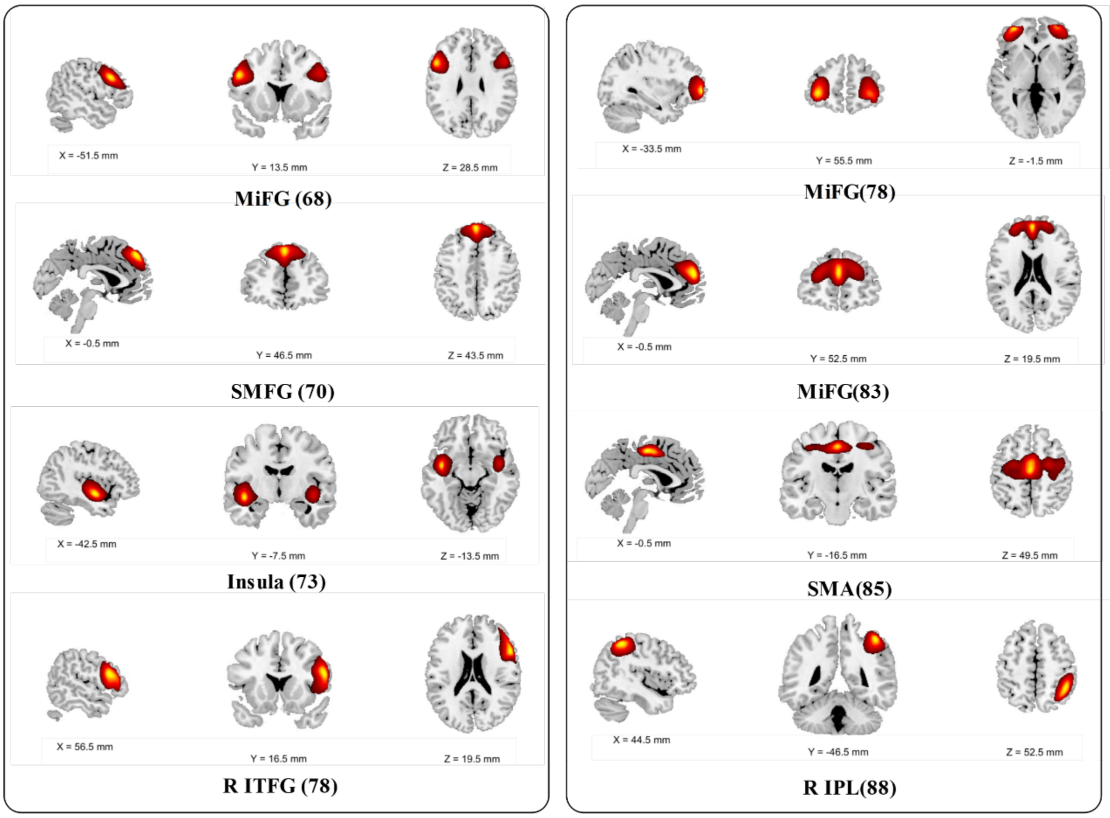

Figure 1-4(2). Spatial maps of intrinsic connectivity networks in cognitive control domain. Sagittal, coronal, and axial slices are shown at the maximal t-statistic for clusters larger than 3 cm^3.

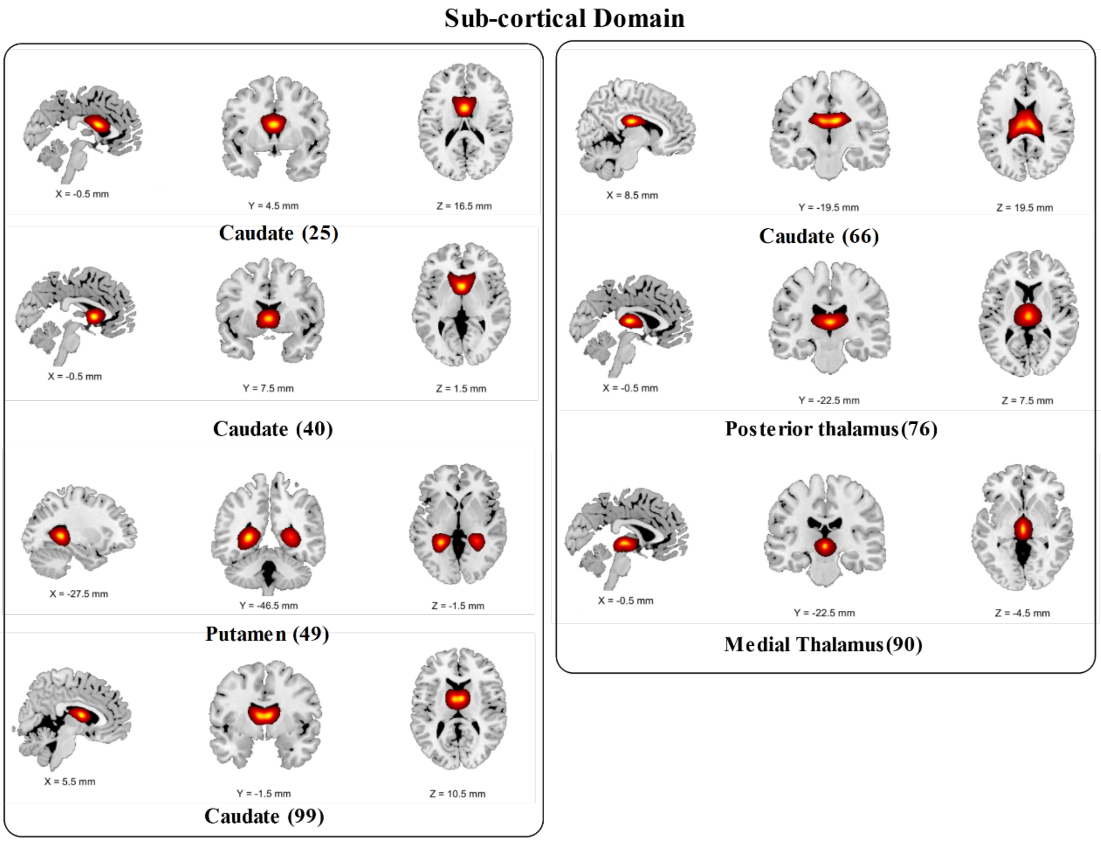

Figure 1-5. Spatial maps of intrinsic connectivity networks in subcortical domain. Sagittal, coronal, and axial slices are shown at the maximal t-statistic for clusters larger than 3 cm^3.

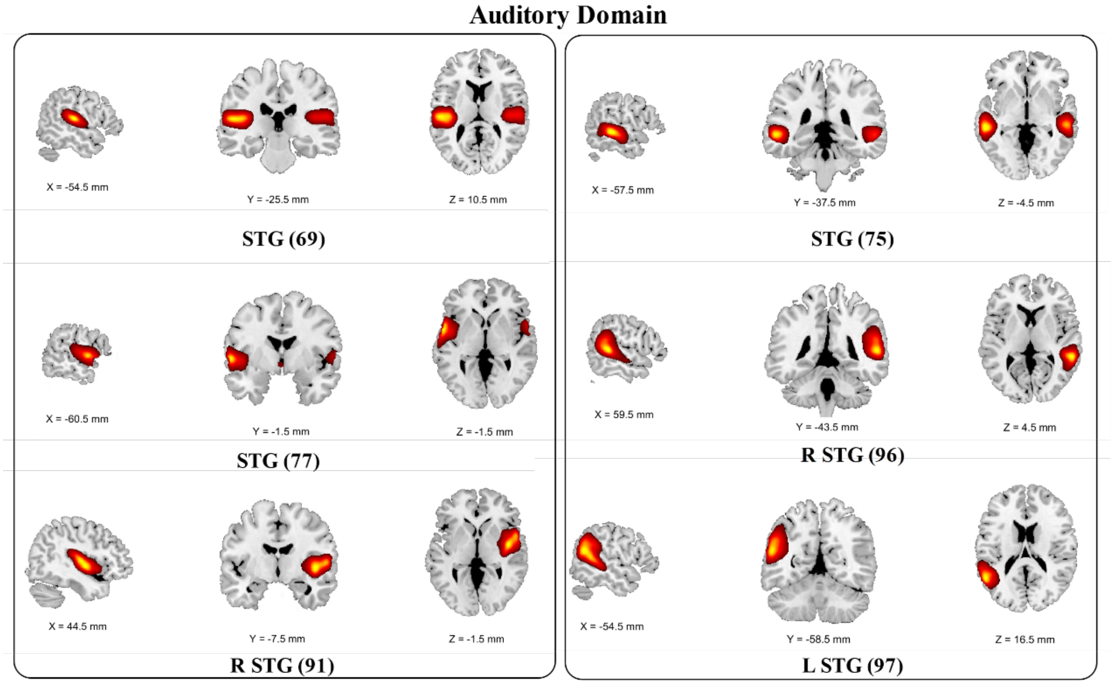

Figure 1-6. Spatial maps of intrinsic connectivity networks in subcortical domain. Sagittal, coronal, and axial slices are shown at the maximal t-statistic for clusters larger than 3 cm^3.
